# Poly[[aqua­tris­(μ-benzene-1,4-dicarboxyl­ato)tricobalt(II)] methanol monosolvate monohydrate]

**DOI:** 10.1107/S1600536811020009

**Published:** 2011-06-04

**Authors:** Hu Zhou, Chao-Xia Chu, Yi-Zhi Li

**Affiliations:** a School of Material Science and Engineering, Jiangsu University of Science and Technology, Zhenjiang 212003, People’s Republic of China; b Coordination Chemistry Institute and the State Key Laboratory of Coordination Chemistry, Nanjing University, Nanjing 210093, People’s Republic of China

## Abstract

The asymmetric unit of the title compound, {[Co_3_(C_8_H_4_O_4_)_3_(H_2_O)]·CH_3_OH·H_2_O}_*n*_, consists of four crystallographically independent Co cations, four benzene-1,4-dicarboxyl­ate (bdc) anions, two water and one methanol solvent mol­ecule. Two of the Co cations and two of the bdc anions are located on centres of inversion, whereas all other atoms are located in general positions. In the crystal, two Co atoms are only fourfold coordinated by three O atoms from three bdc ligands and by one O atom from one coordinated water mol­ecule, while a third Co atom is coordinated by four O atoms from four bdc ligands within a strongly distorted tetra­hedral geometry. The other two Co cations are octa­hedrally coordinated by six O atoms from six bdc anions. The Co cations are linked by the bdc anions into a three-dimensional framework. From this arrangement, cavities are formed in which additional methanol and water mol­ecules are embedded.

## Related literature

For related structures, see: Rosi *et al.* (2005 ▶); Devic *et al.* (2005 ▶); Humphrey *et al.* (2007 ▶); Luo *et al.* (2007 ▶, 2008 ▶). For general background to benzene-1,4-dicarb­oxy­lic acid (H_2_bdc), see: Férey *et al.* (2005 ▶); Rosi *et al.* (2003 ▶). For background to metal-organic frameworks (MOFs), see: Long & Yaghi (2009 ▶).
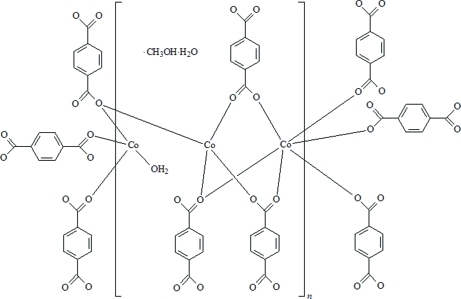


## Experimental

### 

#### Crystal data

[Co_3_(C_8_H_4_O_4_)_3_(H_2_O)]·CH_4_O·H_2_O
*M* *_r_* = 737.20
Triclinic, 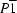

*a* = 9.8456 (11) Å
*b* = 12.0753 (15) Å
*c* = 13.0039 (16) Å
α = 91.842 (2)°
β = 100.925 (1)°
γ = 101.745 (1)°
*V* = 1482.2 (3) Å^3^
*Z* = 2
Mo *K*α radiation
μ = 1.73 mm^−1^
*T* = 291 K
0.30 × 0.26 × 0.24 mm

#### Data collection

Bruker SMART APEX CCD diffractometer
Absorption correction: multi-scan (*SADABS*; Bruker, 2004 ▶) *T*_min_ = 0.682, *T*_max_ = 0.746
11744 measured reflections
5751 independent reflections
4241 reflections with *I* > 2σ(*I*)
*R*_int_ = 0.031

#### Refinement

*R*[*F* ^2^ > 2σ(*F* ^2^)] = 0.051
*wR*(*F* ^2^) = 0.110
*S* = 1.04
5751 reflections
392 parameters
H-atom parameters constrained
Δρ_max_ = 0.36 e Å^−3^
Δρ_min_ = −0.70 e Å^−3^

### 

Data collection: *SMART* (Bruker, 2004 ▶); cell refinement: *SAINT* (Bruker, 2004 ▶); data reduction: *SAINT*; program(s) used to solve structure: *SHELXS97* (Sheldrick, 2008 ▶); program(s) used to refine structure: *SHELXL97* (Sheldrick, 2008 ▶); molecular graphics: *DIAMOND* (Brandenburg, 2006 ▶); software used to prepare material for publication: *SHELXTL* (Sheldrick, 2008 ▶).

## Supplementary Material

Crystal structure: contains datablock(s) I, global. DOI: 10.1107/S1600536811020009/nc2230sup1.cif

Structure factors: contains datablock(s) I. DOI: 10.1107/S1600536811020009/nc2230Isup2.hkl

Additional supplementary materials:  crystallographic information; 3D view; checkCIF report

## Figures and Tables

**Fig. 1.**
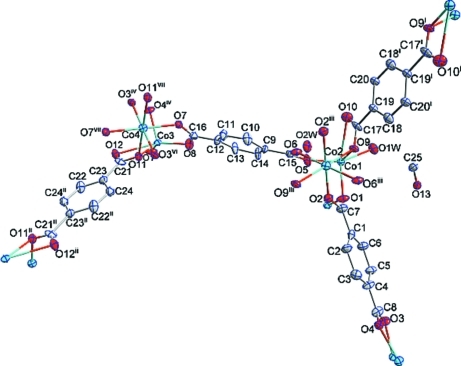
ORTEP diagram of the title compound. Displacement ellipsoids are drawn at 30% probability level. Hydrogen atoms have been omitted for clarity. Symmetry codes: (i) -x, -y + 1, -z + 1; (ii) -x + 3, -y + 2, -z; (iii) -x + 1, -y + 1, -z + 1; (iv) x, y + 1, z - 1; (v) -x + 2, -y + 2, -z + 1; (vi) -x + 2, -y + 1, -z + 1; (vii) -x + 2, -y + 2, -z.

## supplementary crystallographic information

### Comment

Recently, research on metal-organic frameworks (MOFs) has become of increasing interest (Long *et al.*, 2009). In this context compounds based on dicarboxylate ligands are of special importance (Férey *et al.*, 2005; Rosi *et al.*, 2003). In our own investigations we tried to prepare new MOFs based on benzene-1,4-dicarboxylic acid (H_2_bdc) and amines as ligands. Within this project we have reacted CoCl_2_.6H_2_O with H_2_bdc and *N*,*N'*-bis(4-pyridylmethylidine)-1,4-phenylenediamine and we have obtained crystals of the title compound by accident.

The title compound is a three-dimensional framework the form hex type (Rosi *et al.*, 2005) built from Co cations that are linked by bdc anions. From this arrangement cavities are formed, that are filled with additional uncoordinated methanol and water molecules. Altogether there are four crystallographically independent Co cations of which two (Co2 and Co4) are located on centers of inversion. Co1 is coordinated by three oxygen atoms from three bdc ligands and one oxygen atom from one coordinated water molecule within a strongly distorted tetrahedron. Co3 is also tetrahedrally coordinated but connected only to four oxygen atoms from four bdc ligands. In contrast Co2 and Co4 are 6-fold coordinated by six oxygen atoms from bdc anions within slightly distorted octahedra. Related complexes have been reported recently (Devic *et al.*, 2005; Humphrey *et al.*, 2007; Luo *et al.*, 2007; Luo *et al.*, 2008).

### Experimental

A mixture of CoCl_2_.6H_2_O (0.0238 g, 0.1 mmol), benzene-1,4-dicarboxylic acid (0.0166 g, 0.1 mmol), *N*,*N'*-bis(4-pyridylmethylidine)-1,4-phenylenediamine (0.0290 g, 0.1 mmol) combined with 5 ml mixed solvent (DMF:CH_3_OH = 1:1) was stirred for 20 min at room temperature. Afterwards the solution was heated in a 25 ml Teflon-lined stainless-steel vessel at 140 *^o^*C for 72 h under autogenous pressure. Slow cooling of the resulting solution to room temperature at the rate of 10 *^o^*C.h^-1^ afforded purple block-shaped crystals suitable for single-crystal X-ray structure analysis. Yield based on CoCl_2_.6H_2_O: 35%.

### Refinement

All non-hydrogen atoms were refined with anisotropic thermal parameters. The C-H H atoms were calculated in idealized positions with C—H = 0.95 or 0.96 Å and included in the refinement in a riding mode with *U*_iso_ for H assigned as 1.2 or 1.5 times *U*_eq_ of the attached atoms. The H atoms bound to oxygen atoms from coordinated and crystallized water molecules were located from difference maps and refined as riding, with O - H restraint (O - H = 0.96 or 0.85 Å), and with *U*_iso_(H) = 1.2*U*_eq_(O).

### Crystal data

**Table:**

[Co_3_(C_8_H_4_O_4_)_3_(H_2_O)]·CH_4_O·H_2_O	*Z* = 2
*M**_r_* = 737.20	*F*(000) = 742
Triclinic, *P*1	*D*_x_ = 1.652 Mg m^−^^3^
Hall symbol: -P 1	Mo *K*α radiation, λ = 0.71073 Å
*a* = 9.8456 (11) Å	Cell parameters from 3571 reflections
*b* = 12.0753 (15) Å	θ = 2.2–24.1°
*c* = 13.0039 (16) Å	µ = 1.73 mm^−^^1^
α = 91.842 (2)°	*T* = 291 K
β = 100.925 (1)°	Block, purple
γ = 101.745 (1)°	0.30 × 0.26 × 0.24 mm
*V* = 1482.2 (3) Å^3^	

### Data collection

**Table:**

Bruker SMART APEX CCD diffractometer	5751 independent reflections
Radiation source: sealed tube	4241 reflections with *I* > 2σ(*I*)
graphite	*R*_int_ = 0.031
φ and ω scans	θ_max_ = 26.0°, θ_min_ = 1.6°
Absorption correction: multi-scan (*SADABS*; Bruker, 2004)	*h* = −11→12
*T*_min_ = 0.682, *T*_max_ = 0.746	*k* = −14→14
11744 measured reflections	*l* = −15→16

### Refinement

**Table:**

Refinement on *F*^2^	Primary atom site location: structure-invariant direct methods
Least-squares matrix: full	Secondary atom site location: difference Fourier map
*R*[*F*^2^ > 2σ(*F*^2^)] = 0.051	Hydrogen site location: inferred from neighbouring sites
*wR*(*F*^2^) = 0.110	H-atom parameters constrained
*S* = 1.04	*w* = 1/[σ^2^(*F*_o_^2^) + (0.05*P*)^2^ + 1.22*P*] where *P* = (*F*_o_^2^ + 2*F*_c_^2^)/3
5751 reflections	(Δ/σ)_max_ < 0.001
392 parameters	Δρ_max_ = 0.36 e Å^−^^3^
0 restraints	Δρ_min_ = −0.70 e Å^−^^3^

### Special details

**Table:**

Geometry. All e.s.d.'s (except the e.s.d. in the dihedral angle between two l.s. planes) are estimated using the full covariance matrix. The cell e.s.d.'s are taken into account individually in the estimation of e.s.d.'s in distances, angles and torsion angles; correlations between e.s.d.'s in cell parameters are only used when they are defined by crystal symmetry. An approximate (isotropic) treatment of cell e.s.d.'s is used for estimating e.s.d.'s involving l.s. planes.
Refinement. Refinement of *F*^2^ against ALL reflections. The weighted *R*-factor *wR* and goodness of fit *S* are based on *F*^2^, conventional *R*-factors *R* are based on *F*, with *F* set to zero for negative *F*^2^. The threshold expression of *F*^2^ > σ(*F*^2^) is used only for calculating *R*-factors(gt) *etc*. and is not relevant to the choice of reflections for refinement. *R*-factors based on *F*^2^ are statistically about twice as large as those based on *F*, and *R*- factors based on ALL data will be even larger.

### Fractional atomic coordinates and isotropic or equivalent isotropic displacement parameters (Å^2^)

**Table:**

	*x*	*y*	*z*	*U*_iso_*/*U*_eq_	
C1	0.7655 (4)	0.4662 (3)	0.7910 (3)	0.0345 (9)	
C2	0.7907 (5)	0.3680 (4)	0.7456 (4)	0.0454 (11)	
H2	0.7628	0.3508	0.6719	0.054*	
C3	0.8574 (5)	0.2946 (4)	0.8090 (4)	0.0419 (10)	
H3	0.8717	0.2262	0.7790	0.050*	
C4	0.9021 (5)	0.3234 (3)	0.9161 (3)	0.0398 (10)	
C5	0.8818 (5)	0.4247 (4)	0.9588 (4)	0.0485 (12)	
H5	0.9157	0.4461	1.0315	0.058*	
C6	0.8129 (5)	0.4934 (4)	0.8958 (3)	0.0426 (10)	
H6	0.7980	0.5615	0.9261	0.051*	
C7	0.6950 (5)	0.5449 (4)	0.7247 (4)	0.0412 (10)	
C8	0.9731 (5)	0.2503 (3)	0.9811 (4)	0.0422 (11)	
C9	0.7203 (4)	0.8267 (3)	0.4169 (3)	0.0301 (8)	
C10	0.6962 (5)	0.7946 (4)	0.3063 (4)	0.0505 (12)	
H10	0.6323	0.7266	0.2758	0.061*	
C11	0.7716 (5)	0.8688 (4)	0.2457 (4)	0.0429 (10)	
H11	0.7599	0.8486	0.1728	0.052*	
C12	0.8616 (5)	0.9694 (3)	0.2872 (3)	0.0400 (10)	
C13	0.8770 (5)	1.0004 (4)	0.3927 (4)	0.0501 (12)	
H13	0.9324	1.0721	0.4222	0.060*	
C14	0.8078 (5)	0.9223 (4)	0.4566 (3)	0.0421 (11)	
H14	0.8257	0.9401	0.5302	0.051*	
C15	0.6447 (4)	0.7483 (3)	0.4814 (3)	0.0317 (8)	
C16	0.9373 (4)	1.0442 (4)	0.2203 (3)	0.0375 (9)	
C17	0.2859 (4)	0.6297 (4)	0.5711 (3)	0.0371 (10)	
C18	0.1060 (5)	0.4418 (4)	0.5392 (4)	0.0426 (10)	
H18	0.1803	0.4042	0.5644	0.051*	
C19	0.1364 (4)	0.5612 (4)	0.5314 (3)	0.0356 (9)	
C20	0.0241 (4)	0.6157 (4)	0.4882 (3)	0.0388 (10)	
H20	0.0448	0.6948	0.4795	0.047*	
C21	1.3134 (4)	1.1321 (4)	0.0668 (4)	0.0406 (10)	
C22	1.5397 (5)	1.1132 (4)	−0.0081 (3)	0.0443 (11)	
H22	1.5718	1.1927	−0.0099	0.053*	
C23	1.4218 (5)	1.0740 (3)	0.0374 (3)	0.0378 (9)	
C24	1.3909 (5)	0.9566 (4)	0.0493 (4)	0.0418 (10)	
H24	1.3199	0.9262	0.0871	0.050*	
C25	0.2871 (5)	0.4940 (4)	0.8243 (4)	0.0463 (11)	
H25A	0.1955	0.4495	0.8330	0.069*	
H25C	0.3313	0.4480	0.7826	0.069*	
H25D	0.2728	0.5619	0.7881	0.069*	
Co1	0.53756 (6)	0.71571 (5)	0.67841 (4)	0.03287 (15)	
Co2	0.5000	0.5000	0.5000	0.03250 (18)	
Co3	1.15981 (5)	1.21536 (4)	0.17863 (4)	0.02938 (14)	
Co4	1.0000	1.0000	0.0000	0.03193 (18)	
O1	0.6831 (3)	0.6278 (3)	0.7537 (2)	0.0465 (8)	
O2	0.6588 (3)	0.5129 (3)	0.6183 (2)	0.0415 (7)	
O3	0.9700 (3)	0.1490 (2)	0.9415 (2)	0.0377 (7)	
O4	1.0326 (3)	0.2841 (2)	1.0748 (2)	0.0410 (7)	
O5	0.6481 (3)	0.7837 (3)	0.5886 (3)	0.0476 (8)	
O6	0.5728 (3)	0.6495 (2)	0.4424 (2)	0.0375 (7)	
O7	0.9028 (3)	1.0201 (2)	0.1217 (2)	0.0352 (6)	
O8	1.0366 (3)	1.1244 (3)	0.2642 (2)	0.0488 (8)	
O9	0.3832 (3)	0.5818 (2)	0.6010 (2)	0.0344 (6)	
O10	0.3048 (3)	0.7359 (3)	0.5713 (3)	0.0472 (8)	
O11	1.2079 (3)	1.0796 (2)	0.0968 (2)	0.0378 (7)	
O12	1.3470 (3)	1.2378 (3)	0.0664 (2)	0.0431 (7)	
O13	0.3793 (3)	0.5276 (3)	0.9276 (2)	0.0409 (7)	
H15B	0.4504	0.5936	0.9225	0.049*	
O1W	0.4003 (3)	0.7587 (3)	0.7928 (3)	0.0458 (7)	
H1X	0.3246	0.7899	0.7555	0.055*	
H1Y	0.4577	0.8130	0.8474	0.055*	
O2W	0.6664 (4)	0.9904 (3)	0.6639 (3)	0.0574 (9)	
H2X	0.6605	0.9232	0.6394	0.069*	
H2Y	0.7051	0.9975	0.7287	0.069*	

### Atomic displacement parameters (Å^2^)

**Table:**

	*U*^11^	*U*^22^	*U*^33^	*U*^12^	*U*^13^	*U*^23^
C1	0.035 (2)	0.0287 (19)	0.037 (2)	−0.0018 (16)	0.0105 (17)	0.0053 (16)
C2	0.052 (3)	0.040 (2)	0.049 (3)	0.021 (2)	0.007 (2)	0.010 (2)
C3	0.043 (2)	0.040 (2)	0.042 (2)	0.0108 (19)	0.0044 (19)	−0.0060 (19)
C4	0.043 (2)	0.0248 (19)	0.039 (2)	0.0000 (17)	−0.0150 (19)	0.0038 (17)
C5	0.042 (3)	0.049 (3)	0.051 (3)	0.015 (2)	−0.006 (2)	0.009 (2)
C6	0.046 (3)	0.041 (2)	0.039 (2)	0.014 (2)	−0.001 (2)	0.0028 (19)
C7	0.048 (3)	0.036 (2)	0.039 (2)	0.0143 (19)	−0.0013 (19)	0.0020 (19)
C8	0.042 (2)	0.027 (2)	0.043 (2)	−0.0058 (17)	−0.0123 (19)	0.0003 (18)
C9	0.038 (2)	0.0307 (19)	0.0259 (19)	0.0086 (16)	0.0154 (16)	0.0076 (15)
C10	0.046 (3)	0.049 (3)	0.047 (3)	−0.014 (2)	0.012 (2)	−0.009 (2)
C11	0.039 (2)	0.043 (2)	0.042 (2)	−0.0065 (19)	0.0136 (19)	0.0080 (19)
C12	0.052 (3)	0.030 (2)	0.038 (2)	0.0021 (18)	0.014 (2)	0.0051 (17)
C13	0.039 (3)	0.058 (3)	0.044 (3)	−0.016 (2)	0.017 (2)	−0.006 (2)
C14	0.045 (2)	0.047 (2)	0.029 (2)	−0.0140 (19)	0.0191 (18)	−0.0087 (18)
C15	0.031 (2)	0.033 (2)	0.035 (2)	0.0109 (17)	0.0111 (17)	0.0033 (16)
C16	0.029 (2)	0.043 (2)	0.037 (2)	0.0043 (18)	0.0010 (17)	0.0074 (18)
C17	0.024 (2)	0.047 (2)	0.042 (2)	0.0131 (18)	0.0088 (17)	−0.0194 (19)
C18	0.039 (2)	0.050 (3)	0.040 (2)	0.011 (2)	0.0109 (19)	−0.009 (2)
C19	0.0220 (19)	0.047 (2)	0.040 (2)	0.0097 (17)	0.0076 (16)	0.0106 (18)
C20	0.027 (2)	0.047 (2)	0.045 (2)	0.0127 (18)	0.0089 (18)	0.010 (2)
C21	0.021 (2)	0.046 (3)	0.048 (3)	0.0071 (18)	−0.0085 (18)	−0.012 (2)
C22	0.047 (3)	0.038 (2)	0.040 (2)	−0.009 (2)	0.014 (2)	−0.0141 (19)
C23	0.049 (3)	0.031 (2)	0.039 (2)	0.0132 (18)	0.0160 (19)	0.0091 (17)
C24	0.039 (2)	0.046 (3)	0.041 (2)	0.005 (2)	0.0135 (19)	0.007 (2)
C25	0.052 (3)	0.050 (3)	0.042 (3)	0.020 (2)	0.011 (2)	0.017 (2)
Co1	0.0347 (3)	0.0324 (3)	0.0326 (3)	0.0089 (2)	0.0080 (2)	−0.0003 (2)
Co2	0.0330 (4)	0.0316 (4)	0.0325 (4)	0.0079 (3)	0.0049 (3)	−0.0009 (3)
Co3	0.0267 (3)	0.0323 (3)	0.0271 (3)	0.0056 (2)	0.0015 (2)	0.0005 (2)
Co4	0.0336 (4)	0.0275 (4)	0.0332 (4)	0.0048 (3)	0.0052 (3)	0.0004 (3)
O1	0.0524 (19)	0.0331 (17)	0.0452 (18)	0.0145 (14)	−0.0167 (15)	−0.0057 (14)
O2	0.0440 (17)	0.0429 (17)	0.0382 (16)	0.0121 (14)	0.0057 (13)	0.0069 (13)
O3	0.0389 (16)	0.0433 (16)	0.0391 (16)	0.0201 (13)	0.0150 (13)	0.0105 (13)
O4	0.0444 (17)	0.0395 (16)	0.0288 (15)	0.0059 (13)	−0.0132 (13)	−0.0077 (12)
O5	0.0482 (18)	0.0426 (17)	0.0477 (18)	−0.0097 (14)	0.0222 (15)	−0.0115 (14)
O6	0.0356 (15)	0.0306 (15)	0.0393 (16)	0.0071 (12)	−0.0088 (12)	−0.0015 (12)
O7	0.0272 (14)	0.0441 (16)	0.0321 (16)	0.0020 (12)	0.0061 (12)	0.0048 (12)
O8	0.0491 (19)	0.0430 (18)	0.0435 (18)	−0.0162 (15)	0.0123 (15)	−0.0080 (14)
O9	0.0275 (14)	0.0438 (16)	0.0333 (15)	0.0138 (12)	0.0038 (11)	−0.0045 (12)
O10	0.0519 (19)	0.0427 (18)	0.0460 (18)	0.0149 (15)	0.0051 (15)	−0.0127 (14)
O11	0.0399 (17)	0.0416 (16)	0.0320 (15)	0.0062 (13)	0.0105 (13)	−0.0001 (12)
O12	0.0432 (17)	0.0422 (17)	0.0427 (17)	0.0040 (14)	0.0142 (14)	−0.0160 (14)
O13	0.0421 (17)	0.0412 (17)	0.0441 (17)	0.0105 (13)	0.0165 (14)	0.0129 (13)
O1W	0.0454 (18)	0.0419 (17)	0.0494 (18)	0.0047 (14)	0.0156 (14)	−0.0145 (14)
O2W	0.058 (2)	0.051 (2)	0.053 (2)	−0.0121 (16)	0.0132 (17)	−0.0059 (16)

### Geometric parameters (Å, °)

**Table:**

C1—C6	1.360 (6)	C21—O11	1.239 (5)
C1—C2	1.395 (6)	C21—O12	1.252 (5)
C1—C7	1.491 (6)	C21—C23	1.489 (6)
C2—C3	1.405 (6)	C22—C24^ii^	1.345 (6)
C2—H2	0.9500	C22—C23	1.406 (6)
C3—C4	1.388 (6)	C22—H22	0.9500
C3—H3	0.9500	C23—C24	1.408 (6)
C4—C5	1.393 (6)	C24—C22^ii^	1.345 (6)
C4—C8	1.435 (6)	C24—H24	0.9500
C5—C6	1.372 (6)	C25—O13	1.465 (5)
C5—H5	0.9500	C25—H25A	0.9800
C6—H6	0.9500	C25—H25C	0.9800
C7—O1	1.095 (5)	C25—H25D	0.9800
C7—O2	1.383 (5)	Co1—O5	1.845 (3)
C8—O4	1.262 (5)	Co1—O9	2.062 (3)
C8—O3	1.305 (5)	Co1—O1	2.072 (3)
C9—C14	1.315 (6)	Co1—O1W	2.304 (3)
C9—C10	1.440 (6)	Co2—O2	1.952 (3)
C9—C15	1.468 (5)	Co2—O2^iii^	1.952 (3)
C10—C11	1.404 (6)	Co2—O6	2.024 (3)
C10—H10	0.9500	Co2—O6^iii^	2.024 (3)
C11—C12	1.376 (6)	Co2—O9	2.230 (3)
C11—H11	0.9500	Co2—O9^iii^	2.230 (3)
C12—C13	1.381 (6)	Co3—O4^iv^	1.984 (3)
C12—C16	1.467 (6)	Co3—O8	1.990 (3)
C13—C14	1.431 (6)	Co3—O11	2.107 (3)
C13—H13	0.9500	Co3—O1^v^	2.222 (3)
C14—H14	0.9500	Co4—O3^iv^	2.030 (3)
C15—O6	1.289 (5)	Co4—O3^vi^	2.030 (3)
C15—O5	1.437 (5)	Co4—O7	2.030 (3)
C16—O8	1.260 (5)	Co4—O7^vii^	2.030 (3)
C16—O7	1.271 (5)	Co4—O11	2.201 (3)
C17—O9	1.226 (5)	Co4—O11^vii^	2.201 (3)
C17—O10	1.258 (5)	O1—Co3^v^	2.222 (3)
C17—C19	1.519 (6)	O3—Co4^viii^	2.030 (3)
C18—C20^i^	1.304 (6)	O4—Co3^viii^	1.984 (3)
C18—C19	1.422 (6)	O13—H15B	0.9600
C18—H18	0.9500	O1W—H1X	0.9601
C19—C20	1.434 (5)	O1W—H1Y	0.9600
C20—C18^i^	1.304 (6)	O2W—H2X	0.8501
C20—H20	0.9500	O2W—H2Y	0.8500
			
C6—C1—C2	119.5 (4)	C23—C24—H24	119.4
C6—C1—C7	119.8 (4)	O13—C25—H25A	109.5
C2—C1—C7	120.6 (4)	O13—C25—H25C	109.5
C1—C2—C3	120.0 (4)	H25A—C25—H25C	109.5
C1—C2—H2	120.0	O13—C25—H25D	109.5
C3—C2—H2	120.0	H25A—C25—H25D	109.5
C4—C3—C2	119.1 (4)	H25C—C25—H25D	109.5
C4—C3—H3	120.5	O5—Co1—O9	110.87 (13)
C2—C3—H3	120.5	O5—Co1—O1	96.16 (16)
C3—C4—C5	119.8 (4)	O9—Co1—O1	99.86 (11)
C3—C4—C8	119.5 (4)	O5—Co1—O1W	140.91 (13)
C5—C4—C8	120.6 (4)	O9—Co1—O1W	93.65 (11)
C6—C5—C4	120.0 (4)	O1—Co1—O1W	109.49 (14)
C6—C5—H5	120.0	O2—Co2—O2^iii^	180.000 (1)
C4—C5—H5	120.0	O2—Co2—O6	96.10 (12)
C1—C6—C5	121.5 (4)	O2^iii^—Co2—O6	83.90 (12)
C1—C6—H6	119.3	O2—Co2—O6^iii^	83.90 (12)
C5—C6—H6	119.3	O2^iii^—Co2—O6^iii^	96.10 (12)
O1—C7—O2	120.2 (4)	O6—Co2—O6^iii^	180.0
O1—C7—C1	124.2 (4)	O2—Co2—O9	90.15 (11)
O2—C7—C1	115.2 (4)	O2^iii^—Co2—O9	89.85 (11)
O4—C8—O3	122.1 (4)	O6—Co2—O9	91.77 (11)
O4—C8—C4	119.7 (4)	O6^iii^—Co2—O9	88.23 (11)
O3—C8—C4	118.2 (4)	O2—Co2—O9^iii^	89.85 (11)
C14—C9—C10	120.2 (4)	O2^iii^—Co2—O9^iii^	90.15 (11)
C14—C9—C15	122.6 (4)	O6—Co2—O9^iii^	88.23 (11)
C10—C9—C15	117.2 (4)	O6^iii^—Co2—O9^iii^	91.77 (11)
C11—C10—C9	116.8 (4)	O9—Co2—O9^iii^	180.000 (1)
C11—C10—H10	121.6	O4^iv^—Co3—O8	106.09 (14)
C9—C10—H10	121.6	O4^iv^—Co3—O11	106.51 (11)
C12—C11—C10	122.8 (4)	O8—Co3—O11	97.95 (13)
C12—C11—H11	118.6	O4^iv^—Co3—O1^v^	98.73 (11)
C10—C11—H11	118.6	O8—Co3—O1^v^	121.70 (13)
C11—C12—C13	118.9 (4)	O11—Co3—O1^v^	124.18 (12)
C11—C12—C16	120.6 (4)	O3^iv^—Co4—O3^vi^	180.00 (6)
C13—C12—C16	120.4 (4)	O3^iv^—Co4—O7	93.55 (11)
C12—C13—C14	118.7 (4)	O3^vi^—Co4—O7	86.45 (11)
C12—C13—H13	120.7	O3^iv^—Co4—O7^vii^	86.45 (11)
C14—C13—H13	120.7	O3^vi^—Co4—O7^vii^	93.55 (11)
C9—C14—C13	122.3 (4)	O7—Co4—O7^vii^	180.000 (1)
C9—C14—H14	118.8	O3^iv^—Co4—O11	92.68 (12)
C13—C14—H14	118.8	O3^vi^—Co4—O11	87.32 (12)
O6—C15—O5	119.8 (3)	O7—Co4—O11	91.18 (11)
O6—C15—C9	120.9 (4)	O7^vii^—Co4—O11	88.82 (11)
O5—C15—C9	119.3 (3)	O3^iv^—Co4—O11^vii^	87.32 (12)
O8—C16—O7	124.3 (4)	O3^vi^—Co4—O11^vii^	92.68 (12)
O8—C16—C12	118.2 (4)	O7—Co4—O11^vii^	88.82 (11)
O7—C16—C12	117.5 (4)	O7^vii^—Co4—O11^vii^	91.18 (10)
O9—C17—O10	122.1 (4)	O11—Co4—O11^vii^	180.0
O9—C17—C19	120.4 (4)	C7—O1—Co1	124.3 (3)
O10—C17—C19	117.4 (4)	C7—O1—Co3^v^	132.2 (3)
C20^i^—C18—C19	119.8 (4)	Co1—O1—Co3^v^	93.67 (12)
C20^i^—C18—H18	120.1	C7—O2—Co2	140.1 (3)
C19—C18—H18	120.1	C8—O3—Co4^viii^	135.7 (3)
C18—C19—C20	119.3 (4)	C8—O4—Co3^viii^	130.1 (3)
C18—C19—C17	119.9 (4)	C15—O5—Co1	128.3 (3)
C20—C19—C17	120.8 (4)	C15—O6—Co2	136.0 (3)
C18^i^—C20—C19	120.8 (4)	C16—O7—Co4	138.1 (3)
C18^i^—C20—H20	119.6	C16—O8—Co3	119.8 (3)
C19—C20—H20	119.6	C17—O9—Co1	100.9 (3)
O11—C21—O12	124.7 (4)	C17—O9—Co2	126.7 (3)
O11—C21—C23	122.0 (4)	Co1—O9—Co2	102.39 (11)
O12—C21—C23	113.0 (4)	C21—O11—Co3	98.8 (3)
C24^ii^—C22—C23	123.1 (4)	C21—O11—Co4	127.7 (3)
C24^ii^—C22—H22	118.5	Co3—O11—Co4	101.90 (12)
C23—C22—H22	118.5	C25—O13—H15B	109.3
C22—C23—C24	115.3 (4)	Co1—O1W—H1X	109.6
C22—C23—C21	131.7 (4)	Co1—O1W—H1Y	109.4
C24—C23—C21	112.7 (4)	H1X—O1W—H1Y	109.5
C22^ii^—C24—C23	121.1 (4)	H2X—O2W—H2Y	109.5
C22^ii^—C24—H24	119.4		
			
C6—C1—C2—C3	3.6 (7)	O6^iii^—Co2—O2—C7	−82.0 (4)
C7—C1—C2—C3	179.9 (4)	O9—Co2—O2—C7	6.3 (4)
C1—C2—C3—C4	−2.4 (7)	O9^iii^—Co2—O2—C7	−173.7 (4)
C2—C3—C4—C5	−0.7 (7)	O4—C8—O3—Co4^viii^	17.9 (7)
C2—C3—C4—C8	−178.6 (4)	C4—C8—O3—Co4^viii^	−160.1 (3)
C3—C4—C5—C6	2.5 (7)	O3—C8—O4—Co3^viii^	11.3 (7)
C8—C4—C5—C6	−179.6 (5)	C4—C8—O4—Co3^viii^	−170.8 (3)
C2—C1—C6—C5	−1.8 (7)	O6—C15—O5—Co1	−11.2 (5)
C7—C1—C6—C5	−178.1 (4)	C9—C15—O5—Co1	167.2 (3)
C4—C5—C6—C1	−1.3 (8)	O9—Co1—O5—C15	−5.8 (4)
C6—C1—C7—O1	3.7 (8)	O1—Co1—O5—C15	97.2 (3)
C2—C1—C7—O1	−172.6 (5)	O1W—Co1—O5—C15	−131.1 (3)
C6—C1—C7—O2	176.1 (4)	O5—C15—O6—Co2	−22.8 (6)
C2—C1—C7—O2	−0.2 (6)	C9—C15—O6—Co2	158.9 (3)
C3—C4—C8—O4	169.3 (4)	O2—Co2—O6—C15	−33.7 (4)
C5—C4—C8—O4	−8.6 (7)	O2^iii^—Co2—O6—C15	146.3 (4)
C3—C4—C8—O3	−12.7 (7)	O9—Co2—O6—C15	56.6 (4)
C5—C4—C8—O3	169.4 (4)	O9^iii^—Co2—O6—C15	−123.4 (4)
C14—C9—C10—C11	1.0 (7)	O8—C16—O7—Co4	46.4 (7)
C15—C9—C10—C11	−178.5 (4)	C12—C16—O7—Co4	−129.9 (4)
C9—C10—C11—C12	−1.9 (7)	O3^iv^—Co4—O7—C16	−106.4 (4)
C10—C11—C12—C13	−1.2 (7)	O3^vi^—Co4—O7—C16	73.6 (4)
C10—C11—C12—C16	−179.7 (4)	O11—Co4—O7—C16	−13.6 (4)
C11—C12—C13—C14	5.1 (7)	O11^vii^—Co4—O7—C16	166.4 (4)
C16—C12—C13—C14	−176.4 (4)	O7—C16—O8—Co3	−4.6 (6)
C10—C9—C14—C13	3.0 (7)	C12—C16—O8—Co3	171.7 (3)
C15—C9—C14—C13	−177.5 (4)	O4^iv^—Co3—O8—C16	60.0 (4)
C12—C13—C14—C9	−6.1 (8)	O11—Co3—O8—C16	−49.8 (4)
C14—C9—C15—O6	−171.9 (4)	O1^v^—Co3—O8—C16	171.3 (3)
C10—C9—C15—O6	7.7 (6)	O10—C17—O9—Co1	14.7 (5)
C14—C9—C15—O5	9.8 (6)	C19—C17—O9—Co1	−165.7 (3)
C10—C9—C15—O5	−170.7 (4)	O10—C17—O9—Co2	−99.9 (4)
C11—C12—C16—O8	−167.5 (4)	C19—C17—O9—Co2	79.7 (5)
C13—C12—C16—O8	14.1 (7)	O5—Co1—O9—C17	−90.8 (3)
C11—C12—C16—O7	9.0 (6)	O1—Co1—O9—C17	168.6 (3)
C13—C12—C16—O7	−169.4 (4)	O1W—Co1—O9—C17	58.1 (3)
C20^i^—C18—C19—C20	−3.2 (7)	O5—Co1—O9—Co2	40.87 (17)
C20^i^—C18—C19—C17	175.3 (4)	O1—Co1—O9—Co2	−59.66 (15)
O9—C17—C19—C18	6.7 (6)	O1W—Co1—O9—Co2	−170.18 (12)
O10—C17—C19—C18	−173.7 (4)	O2—Co2—O9—C17	158.8 (4)
O9—C17—C19—C20	−174.8 (4)	O2^iii^—Co2—O9—C17	−21.2 (4)
O10—C17—C19—C20	4.8 (6)	O6—Co2—O9—C17	62.6 (4)
C18—C19—C20—C18^i^	3.2 (7)	O6^iii^—Co2—O9—C17	−117.4 (4)
C17—C19—C20—C18^i^	−175.3 (4)	O2—Co2—O9—Co1	44.79 (13)
C24^ii^—C22—C23—C24	7.7 (8)	O2^iii^—Co2—O9—Co1	−135.21 (13)
C24^ii^—C22—C23—C21	−165.6 (5)	O6—Co2—O9—Co1	−51.32 (12)
O11—C21—C23—C22	172.5 (5)	O6^iii^—Co2—O9—Co1	128.68 (12)
O12—C21—C23—C22	−13.3 (7)	O12—C21—O11—Co3	−15.7 (5)
O11—C21—C23—C24	−0.9 (6)	C23—C21—O11—Co3	157.8 (4)
O12—C21—C23—C24	173.4 (4)	O12—C21—O11—Co4	96.9 (5)
C22—C23—C24—C22^ii^	−7.5 (7)	C23—C21—O11—Co4	−89.6 (5)
C21—C23—C24—C22^ii^	167.0 (4)	O4^iv^—Co3—O11—C21	88.6 (3)
O2—C7—O1—Co1	27.2 (7)	O8—Co3—O11—C21	−161.9 (3)
C1—C7—O1—Co1	−160.8 (3)	O1^v^—Co3—O11—C21	−24.5 (3)
O2—C7—O1—Co3^v^	−109.1 (4)	O4^iv^—Co3—O11—Co4	−43.08 (15)
C1—C7—O1—Co3^v^	63.0 (7)	O8—Co3—O11—Co4	66.39 (14)
O5—Co1—O1—C7	−84.9 (5)	O1^v^—Co3—O11—Co4	−156.19 (11)
O9—Co1—O1—C7	27.6 (5)	O3^iv^—Co4—O11—C21	−58.9 (4)
O1W—Co1—O1—C7	125.1 (4)	O3^vi^—Co4—O11—C21	121.1 (4)
O5—Co1—O1—Co3^v^	64.22 (14)	O7—Co4—O11—C21	−152.6 (4)
O9—Co1—O1—Co3^v^	176.70 (12)	O7^vii^—Co4—O11—C21	27.4 (4)
O1W—Co1—O1—Co3^v^	−85.82 (14)	O3^iv^—Co4—O11—Co3	52.20 (13)
O1—C7—O2—Co2	−53.0 (7)	O3^vi^—Co4—O11—Co3	−127.80 (13)
C1—C7—O2—Co2	134.3 (4)	O7—Co4—O11—Co3	−41.41 (13)
O6—Co2—O2—C7	98.0 (4)	O7^vii^—Co4—O11—Co3	138.59 (13)

Symmetry codes: (i) −*x*, −*y*+1, −*z*+1; (ii) −*x*+3, −*y*+2, −*z*; (iii) −*x*+1, −*y*+1, −*z*+1; (iv) *x*, *y*+1, *z*−1; (v) −*x*+2, −*y*+2, −*z*+1; (vi) −*x*+2, −*y*+1, −*z*+1; (vii) −*x*+2, −*y*+2, −*z*; (viii) *x*, *y*−1, *z*+1.
